# Multi-Object Tracking of Korean Cattle (Hanwoo) in High-Density Barns Using Multi-Temporal Window Repair (MTW-Repair)

**DOI:** 10.3390/ani16193000

**Published:** 2026-09-23

**Authors:** Seong-hun Kim, Dae-Yeong Kang, Mohammad Ismail, Oluwasegun Moses Ogundele, Dasanayaka Lekamalage Vindya Sathsarani Dasanayaka, Hyeon-Tae Kim

**Affiliations:** 1 Department of Smart Farm, Gyeongsang National University, Jinju 52828, Republic of Korea; tjdgnszhd@naver.com (S.-h.K.); dykang@gnu.ac.kr (D.-Y.K.); vindy@gnu.ac.kr (D.L.V.S.D.); 2 Department of Bio-Systems Engineering, Gyeongsang National University, Jinju 52828, Republic of Korea; hossainab17@gmail.com (M.I.); segun@gnu.ac.kr (O.M.O.); 3 Department of Bio-Industrial Machinery Engineering, Institute of Smart Space Agriculture (ISSA), Gyeongsang National University, Jinju 52828, Republic of Korea

**Keywords:** Hanwoo cattle, multi-object tracking, BoT-SORT, trajectory fragmentation, lightweight tracking

## Abstract

Reliable individual tracking supports animal behavior monitoring in precision livestock farming. However, tracking cattle using a single camera in high-density barns is challenging because of frequent occlusion, scale variation, and irregular movement. This study presents a lightweight multi-object tracking framework for densely housed Hanwoo cattle, combining a Hanwoo-trained YOLOv8n detector with appearance-free BoT-SORT association. Frame sampling reduces the detection workload, while an offline Multi-Temporal Window Repair (MTW-Repair) procedure reconnects fragmented trajectories. The framework was evaluated using short annotated video clips and a continuous 215 min barn recording. In the short clips, appearance-free BoT-SORT outperformed ByteTrack while maintaining comparable throughput, and MTW-Repair reduced identity switches across all evaluated frame-sampling intervals. In the long recording, MTW-Repair reduced the total number of generated identities for both trackers. Manual verification of 50 randomly sampled ByteTrack merge events yielded a merge precision of 84.0%. Appearance-based re-identification offered only marginal accuracy gains at a higher computational cost. These findings support the potential of frame sampling and offline trajectory repair for computationally efficient cattle monitoring.

## Simple Summary

This study developed a lightweight and efficient method for tracking individual Hanwoo cattle using a single camera in densely housed barns. The method combines a Hanwoo-trained YOLOv8n detector with bag of tricks for simple online and realtime tracking (BoT-SORT) and a frame-sampling strategy that reduces the number of frames requiring detection. To recover tracking paths that were interrupted by occlusion or missed detections, a Multi-Temporal Window Repair (MTW-Repair) method was introduced. The proposed approach was tested using short video clips and a continuous 215 min barn recording. MTW-Repair consistently reduced identity switches and helped maintain more stable cattle trajectories. Appearance-free tracking performed well while requiring less processing time than appearance-based re-identification. Overall, the results demonstrate that a simple, computationally efficient tracking framework can support long-term individual cattle monitoring in crowded barn environments, with potential applications in precision livestock farming and automated animal welfare and health monitoring.

## 1. Introduction

Animal welfare in precision livestock farming is closely associated with farm profitability and consumer trust. Among the available welfare indicators, behavior is one of the most practical and widely adopted because it directly reflects health status, productivity, and management conditions [1]. However, accurate behavioral interpretation first requires reliable individual tracking, as meaningful temporal behavioral patterns can only be derived when animals are consistently monitored over time [2]. Wearable sensing technologies, including global positioning system (GPS) collars and accelerometers, have been used to monitor livestock movement and behavior [3]. Camera-based approaches provide a complementary means of monitoring animals without attaching devices to them. These approaches estimate animal location and identity directly from images using deep-learning models.

Representative examples include regression-based estimation of dairy cow body coordinates from overhead footage [4], re-identification based on coat-marking uniqueness [5], and depth-based identification for breeds lacking distinctive visual patterns [6]. These studies collectively demonstrate the potential of camera-based monitoring as a practical alternative to physical sensors in livestock environments.

Previous vision-based livestock studies have examined dairy cattle with distinctive coat patterns, particularly Holstein cattle, whose black-and-white markings provide visual cues for individual identification [7,8]. In contrast, Hanwoo cattle have largely uniform brown coats without distinctive markings. These less distinctive appearance cues, together with body overlap, illumination variation, and frequent occlusion in narrow, high-density barns, pose challenges for individual tracking [9,10]. Moreover, overhead single-camera monitoring introduces additional difficulties, including scale variation caused by viewpoint changes, irregular animal movements, and identity ambiguity among visually similar individuals, which collectively increase association errors during long-term tracking.

As deep-learning techniques have advanced, individual cattle recognition has been extensively investigated [5,6]. However, comparatively limited attention has been directed toward simultaneously tracking multiple animals under crowded conditions. Many existing studies rely on simplified experimental scenarios that restrict animal movement. For instance, corridor-based setups, where cattle pass individually, facilitate recognition but do not represent practical barn environments and remain difficult to scale for long-duration monitoring. Multi-animal tracking in unconstrained video is substantially more challenging due to frequent occlusion, scale variation, and irregular movement patterns [11]. A previous study [12] targeting dense Hanwoo cattle environments integrated a scale-adaptive appearance model, a non-linear Kalman filter, and an occlusion-recovery module within a detection-based tracking framework, achieving high data-association performance. However, the appearance feature extractor introduced considerable computational costs, and the authors indicated that its advantages may diminish when inter-individual visual differences are limited. This highlights a fundamental trade-off between identity robustness and computational efficiency in dense livestock tracking.

A common strategy reported in the literature for improving tracking performance is the use of multi-camera systems or appearance-, pattern-, and depth-based identification mechanisms, often combined with dedicated feature-extraction networks [5,6,12]. Although these mechanisms improve inter-individual discrimination, they also introduce considerable computational complexity. Since long-duration monitoring requires continuous analysis of video streams, this computational burden represents a major barrier to practical deployment [11]. As the monitoring duration increases, the primary challenge shifts from single-frame detection accuracy to processing efficiency and trajectory continuity. Particularly in dense environments, identity switches and association errors can accumulate over time, even in motion-based tracking approaches [13,14]. Nevertheless, few studies have achieved stable long-duration tracking using a single camera without relying on appearance cues, representing an important research gap because scalability, simplicity, and computational efficiency are critical requirements for practical on-farm deployment.

This study addresses this gap by proposing a lightweight single-camera framework for tracking densely housed Hanwoo cattle. The framework investigates frame sampling for computational efficiency and introduces an offline Multi-Temporal Window Repair (MTW-Repair) procedure to reconnect fragmented tracklets. Unlike learned tracklet-linking approaches such as AFLink [15] and Split-and-Connect [16], MTW-Repair uses three gap-dependent temporal windows with explicit distance, implied-speed, and bounding-box size-ratio constraints, without requiring a trained linking model. The framework was evaluated using short annotated video clips and approximately 215 min of continuous barn footage to assess tracking performance, computational efficiency, and trajectory repair.

## 2. Materials and Methods

### 2.1. Description of the Dataset

Footage was collected at a commercial Hanwoo cattle farm in Anseong, Republic of Korea, using a fixed ceiling-mounted closed-circuit television (CCTV) camera. The camera position remained unchanged throughout the recording period, with a frame rate of approximately 13 frames per second (fps). The monitored barn measured 7 m × 7 m, with a room height of 5.1 m, and the camera was mounted at an elevation of 3.5 m. The actual herd size remained constant at seven animals across all recordings. All animals were male Hanwoo cattle. Four source recordings (A–D), obtained on different dates, were used for detector training and tracking evaluation. Recording A, captured on 25 July with a duration of approximately 5 h 40 min, was used exclusively for detector training (Figure 1). A total of 1000 images were extracted and manually annotated with Hanwoo cattle bounding boxes. To improve robustness against illumination variation, partial occlusion, and environmental noise, three augmentation techniques (cutout, shadow, and night simulation) were applied to the original image set. After augmentation, 58 mislabeled or low-quality samples were removed through manual inspection. The final detection dataset, comprising 3942 images after augmentation, contained a total of 26,335 annotated cattle instances (training and validation sets combined), corresponding to an average of approximately 6.7 instances per image. This density reflects the high stocking density of the barn environment, in which most of the seven animals were visible within a single frame. The final detection dataset was divided into training and validation sets using an 80:20 ratio. The dataset was split at the source-image level, and all augmented variants derived from the same source image were retained in the same subset to prevent information leakage between the training and validation sets.

Tracking evaluation was conducted using Recordings B–D, which were independent of the detector training data. Recording B, captured on 7 July and lasting approximately 3 h 35 min, was used for long-duration tracking evaluation. A continuous segment of approximately 215 min (168,328 frames) was analyzed; however, frame-level ground truth (GT) was not available for the entire recording. Recording C was also captured on 7 July, while Recording D was captured on 30 July. Thus, Recordings B and C were obtained on the same date but represent separate recording sessions. Recording C, also captured on 7 July, lasted approximately 1 h, while Recording D, captured on 30 July, lasted approximately 6 h. For short-clip evaluation, four approximately 1 min annotated segments, totaling approximately 4 min, were extracted: one from Recording B, two from Recording C, and one from Recording D. Each segment included per-identity bounding-box annotations in the standard multi-object tracking (MOT) format, with the true herd size maintained at seven animals. These frame-level annotations enabled direct calculation of standard MOT metrics, whereas the long-duration segment from Recording B was evaluated using the surrogate measures described in Section 2.5. Weather conditions were not recorded because the study focused on image-based detection and tracking.

### 2.2. Selection of Detectors

Two object detectors, YOLOv8n [17] and YOLOv11n [18], were evaluated using the Hanwoo cattle detection dataset. Both models were trained for 100 epochs with an input resolution of 640 × 640 pixels. Nano-scale (n) variants were selected to support a lightweight tracking pipeline capable of processing several hours of video footage with near real-time efficiency. These models reduce parameter complexity and computational requirements while maintaining sufficient detection accuracy for Hanwoo cattle under overhead-view conditions, where scale variation is relatively constrained. YOLOv8n and YOLOv11n represent successive generations of real-time object detectors and were compared to assess whether improvements in standalone detection capability translate into enhanced tracking performance.

Both models were initialized with COCO-pretrained weights and fine-tuned for 100 epochs at an input resolution of 640 × 640 pixels, with a batch size of 16, an initial learning rate (lr0) of 0.01, and optimizer = auto (automatic SGD/AdamW selection), using the Hanwoo cattle detection dataset. Training used the Ultralytics implementation with a fixed random seed (0) for deterministic training. Data augmentation included HSV color jitter (h = 0.015, s = 0.7, v = 0.4), translation (0.1), scaling (0.5), horizontal flipping (*p* = 0.5), mosaic augmentation (*p* = 1.0), random erasing (*p* = 0.4), and RandAugment policies. Vertical flipping was disabled given the fixed overhead camera orientation. Inference speed (FPS) was measured on a synthetic 1 × 3 × 640 × 640 input tensor using 15 untimed warm-up iterations followed by 200 timed iterations, excluding video I/O and pre-/post-processing on real frames. All experiments in this study, including detector training, tracker inference, and MTW-Repair processing, were conducted on a Mac mini (Apple Incorporated, Cupertino, CA, USA), equipped with an Apple M4 chip and 32 GB of unified memory, running macOS, using Python 3.9.6, PyTorch 2.8.0 with the Apple MPS backend, Ultralytics 8.4.22, and BoxMOT 10.0.84 for tracker implementations.

### 2.3. Comparison and Selection of Trackers

Five trackers were evaluated under identical conditions: ByteTrack, Observation-centric simple online and realtime tracking (OC-SORT), BoT-SORT with appearance, BoT-SORT without appearance, and StrongSORT. These trackers represent different design strategies within the tracking-by-detection framework. ByteTrack is a widely adopted baseline that leverages low-confidence detections for association [13]. OC-SORT is a motion-based tracker that applies observation-centric Kalman filter correction [19], making it suitable for non-linear and irregular motion patterns. BoT-SORT integrates an improved motion model with camera-motion compensation [14], while its appearance module can be enabled or disabled, allowing the isolated evaluation of appearance information within the same tracking framework. StrongSORT is a representative appearance-based tracker in the deep simple online and realtime tracking (DeepSORT) family that relies heavily on appearance embeddings for identity association [15].

To ensure a fair comparison, all trackers were provided with identical detection inputs. The detection confidence threshold was fixed at 0.25, and the frame-rate parameter was set to 13 fps. Tracker comparisons were conducted using the four short ground-truth segments, while long-duration performance was assessed using total identity counts, predicted trajectory durations, per-window merge counts, and manual verification of sampled merge decisions. Based on these evaluations, appearance-free BoT-SORT was selected as the final tracker. ByteTrack was retained as a reference baseline throughout the study because of its widespread adoption and its usefulness for evaluating the effectiveness of MTW-Repair on a tracker prone to trajectory fragmentation.

For long-duration experiments using stride-3 sampling, detection confidence thresholds were adjusted according to the recommended operating points of each tracker: 0.1 for ByteTrack, which is designed to exploit low-confidence detections during association, and 0.25 for BoT-SORT. All reported effects of MTW-Repair were obtained from before-and-after comparisons within the same tracker and confidence setting; therefore, the difference in confidence thresholds does not influence the assessment of MTW-Repair itself. The two BoT-SORT configurations (appearance enabled and disabled) were further used as a ReID ablation study to isolate the contribution of appearance-based re-identification. The appearance model employed was omni-scale network (OSNet) (osnet_x0_25_msmt17), which was pretrained on person re-identification data and not fine-tuned for cattle tracking [20].

### 2.4. Proposed Framework (Frame Sampling, MTW-Repair, and Interpolation)

The proposed lightweight tracking pipeline consists of three sequential stages: frame sampling, linear interpolation, and trajectory repair using MTW-Repair (Figure 2). First, stride-3 frame sampling processes one out of every three frames for detection and tracking, reducing the detection workload by approximately two-thirds. Second, linear interpolation restores predictions at frames omitted by stride sampling. Third, MTW-Repair reconnects fragmented trajectories using temporal-window constraints. For the short ground-truth segments, interpolation was applied prior to MTW-Repair so that fragmented tracklets first had gaps filled by synthetic detections before merge candidates were evaluated. For the long-duration recording, interpolation was retained when calculating total identity counts and the distribution of accepted MTW-Repair merges. However, the active-identity measure was excluded from the final evaluation because interpolated detections distorted the number of simultaneously active identities, as detailed in Section 3.4. Total ID counts for the long-duration recording were computed following interpolation-then-MTW-Repair processing of the stride-3 tracklets, consistent with the short-GT pipeline.

MTW-Repair identifies possible connections between fragmented tracklets using temporal gap, spatial distance, implied motion speed, and bounding-box size variation. Three temporal windows (short, medium, and long) are defined according to the duration of the gap between tracklets. As the temporal gap increases, a larger absolute spatial displacement is permitted, whereas the implied-speed and bounding-box size-ratio constraints become more conservative to reduce incorrect long-range associations. Candidate pairs that satisfy the window-specific conditions are ranked using a merge score combining distance, temporal gap, and size variation, and merging is performed using a one-to-one matching strategy. Because MTW-Repair operates solely on tracker-generated trajectories, it is independent of the underlying tracking algorithm. The merge score is calculated using Equation (1).

(1) $$score=\frac{d}{D}+0.5\times \frac{g}{G_{x}}+20\times \left|log\left(r\right)\right|$$

where d represents the Euclidean distance between the bottom-center point of the final bounding box of the preceding tracklet and that of the first bounding box of the candidate successor tracklet, D represents the image diagonal, g represents the temporal gap between tracklets, $G_{x}$ represents the maximum temporal gap considered for repair, and r represents the ratio between the bounding-box areas at the start of the successor tracklet and the end of the preceding tracklet. The spatial distance and temporal gap are normalized by the image diagonal and the maximum temporal gap, respectively, making all terms in Equation (1) dimensionless. The weighting coefficients were retained from the original formulation and applied to the fixed-camera configuration used in this study. Lower scores indicate higher merging priority. A greedy one-to-one matching rule was applied so that each preceding and successor tracklet could participate in at most one accepted merge. The greedy strategy does not necessarily produce a globally optimal assignment because candidate pairs are selected sequentially by merge score. However, window-specific spatial, speed, and size-ratio constraints restrict the feasible associations, making the greedy approach computationally efficient for offline repair. A globally optimal method, such as the Hungarian algorithm, could be evaluated in future work. Although normalization reduces sensitivity to image resolution and frame rate, cross-camera generalizability was not evaluated and requires validation with different camera configurations.

All temporal gaps in MTW-Repair were expressed using the original 13 fps frame index. The base short, medium, and long temporal windows were defined at stride-1 as 1–13, 14–65, and 66–260 frames, respectively. For a stride value of size ratio, the implementation boundaries were multiplied by s to accommodate the increased spacing between processed observations. Accordingly, the temporal coverage of each window increased with the stride value. The corresponding boundaries were 2–26, 28–130, and 132–520 frames at stride-2; 3–39, 40–195, and 196–780 frames at stride-3; and 5–65, 70–325, and 330–1300 frames at stride-5. Distance, implied-speed, and size-ratio thresholds were held constant across stride settings. Table 1 and Figure 3 present the base stride-1 parameters.

MTW-Repair can be considered an offline global-linking approach that reconnects fragmented tracklets after online tracking. Existing methods, including global information-aware object tracker (GIAOTracker) [21], strong simple online and realtime tracking (StrongSORT)-based appearance-free linker (AFLink) [15] and object-centric association with feature interaction (OCAFI) [22], and Split-and-Connect [16], belong to the same general category. However, MTW-Repair differs from these approaches by (i) applying multiple gap-adaptive temporal windows with progressively adjusted constraints, (ii) relying exclusively on hand-crafted spatio-temporal rules without learned models, and (iii) being validated using 215 min of continuous livestock monitoring footage.

Linear interpolation was used to restore predictions at frames skipped during stride sampling. For the short ground-truth segments, interpolation was applied before MTW-Repair, enabling frame-level comparison with the full-frame ground truth. The use of interpolation in the long-duration pipeline and its treatment in the surrogate evaluation are described above and in Section 3.4. The complete MTW-Repair procedure is summarized in Algorithm 1.
**Algorithm 1**. Multi-Temporal Window RepairInput: Tracker output containing fragmented trackletsOutput: Repaired trajectories1.Extract all tracklets from the tracking result.2.For each terminated tracklet *Ti*, find candidate successor tracklets *Tj* that start after *Ti* ends.3.Compute the temporal gap and assign the pair to a short, medium, or long window.4.Compute the spatial distance, implied speed, and size-ratio difference.5.Discard candidate pairs that violate the window-specific constraints.6.Compute the merge score for the remaining candidates.7.Sort the candidates by score and merge valid pairs in a one-to-one manner (greedy matching).8.Return the repaired trajectories.

### 2.5. Evaluation Metrics

Tracking performance was evaluated using higher order tracking accuracy (HOTA) [23], association accuracy (AssA) [23], the identification F1 score (IDF1) [24], and multi-object tracking accuracy (MOTA) [25], as defined in Equations (2)–(6). MOTA summarizes false positives, false negatives, and identity switches across frames. Its identity-switch penalty does not directly account for the duration of an incorrect identity assignment, and the metric can be dominated by detection errors. IDF1 evaluates identity consistency through a global one-to-one assignment between predicted and ground-truth trajectories over the evaluated sequence, allowing trajectories to remain unmatched. It is the harmonic mean of identity precision and identity recall under this assignment. AssA measures temporal association consistency by averaging the association Jaccard score over matched detections. HOTA combines detection and association accuracy through their geometric mean at each localization threshold, followed by averaging across thresholds. Because MTW-Repair reconnects tracklets without changing the underlying detection boxes, its effect was interpreted primarily using association-related measures, particularly AssA and identity switches (IDSW), together with HOTA.

IDF1, MOTA, and AssA are defined in Equations (2), (3) and (4), respectively. Equation (5) defines HOTA at a given localization threshold, and Equation (6) averages HOTA across the evaluated thresholds.

(2) $$IDF1=\frac{|IDTP|\;}{\left(\left|IDTP\right|+0.5\left|IDFN\right|+0.5|IDFP|\right)}$$

(3) $$MOTA=1-\frac{\left(\left|FN\right|+\left|FP\right|+\left|IDSW\right|\right)}{\left|gtDet\right|}$$

(4) $$AssA=\frac{1}{|TP|}\sum_{c\in TP}\frac{\left|TPA(c)\right|}{\left|TPA\left(c\right)\right|+\left|FNA\left(c\right)\right|+|FPA(c)|}$$

(5) $${HOTA}_{\alpha }=\sqrt{{DetA}_{\alpha }\times {AssA}_{\alpha }}$$

(6) $$HOTA\approx \frac{1}{19}\sum_{\alpha =0.05}^{0.95}{HOTA}_{\alpha }$$

where IDTP is identity true positive, IDFN is identity false negative, IDFP is identity false positive, FP is false positive, FN is false negative, IDSW is identity switch, gtDet is Ground Truth Detections, TPA is True Positive Association, FNA is false negative association, FPA is false positive association, and ${DetA}_{\alpha }$ is detection accuracy.

For the short ground-truth segments, HOTA, AssA, MOTA, IDF1, IDSW, and the number of generated identities were calculated using TrackEval [23]. Because complete ground truth was unavailable for the long-duration recording, performance was assessed using surrogate measures based on total identity counts and per-window merge counts, together with manual verification of a sample of merge decisions. For manual verification, 50 merge events were randomly sampled from the long-duration stride-3 ByteTrack output. ByteTrack was selected because it produced more merge events than BoT-SORT under this condition. Each sampled merge was visually classified as correct if the two tracklets represented the same animal and incorrect otherwise. Merge precision was calculated as the proportion of correct merges among the sampled events. Wilson 95% confidence intervals (CI) were calculated for the overall and window-specific merge precision estimates. Total identity count reflects cumulative trajectory fragmentation over the full recording, and its reduction after MTW-Repair provides a ground-truth-free indicator of improved trajectory continuity. This is conceptually related to the Fragmentation (FM) metric used in the MOT Challenge benchmark, which counts how many times a ground-truth trajectory is interrupted.

## 3. Results

### 3.1. Detection Performance

Detection performance of the two YOLO-based models is summarized in Table 2. Both detectors achieved precision and recall values above 0.96, indicating reliable Hanwoo cattle detection under the barn conditions evaluated. Based on the standalone detection metrics, YOLOv11n slightly outperformed YOLOv8n, achieving a precision of 0.977, a recall of 0.965, and an mAP50 of 0.986, compared with 0.971, 0.961, and 0.981 for YOLOv8n, respectively. YOLOv11n also showed a higher inference speed, reaching 133.5 FPS compared with 91.7 FPS for YOLOv8n. However, when integrated with the tracking framework, YOLOv8n achieved superior tracking performance and was therefore selected as the final detector (Figure 4).

### 3.2. Tracker Comparison and the Role of Appearance Information

Table 3 compares five tracker configurations using YOLOv8n and YOLOv11n across the four short annotated segments. YOLOv8n produced higher HOTA, MOTA, and IDF1 values than YOLOv11n for each corresponding tracker configuration. With YOLOv8n, ByteTrack, OC-SORT, and appearance-free BoT-SORT achieved HOTA values of 71.0–72.3 and processing speeds of 71.3–77.1 FPS. Enabling appearance information in BoT-SORT increased HOTA from 72.3 to 72.5 and IDF1 from 79.5 to 80.1, while reducing IDSW from 24 to 19 and processing speed from 71.3 to 30 FPS. StrongSORT achieved a HOTA of 71.3 at 12 FPS. With YOLOv11n, enabling appearance information in BoT-SORT increased HOTA from 69.5 to 70.4 and IDF1 from 75.3 to 76.5, while IDSW increased from 16 to 19 and processing speed decreased from 70.0 to 30.6 FPS. YOLOv8n with appearance-free BoT-SORT was selected for the subsequent experiments.

Table 3 demonstrates that the tracking strategy had a greater influence on overall performance than the inclusion of appearance information. Using YOLOv8n, motion-based approaches, including ByteTrack, OC-SORT, and appearance-free BoT-SORT, achieved HOTA values ranging from 71.0 to 72.3 and IDF1 values ranging from 78.7 to 79.5 while maintaining processing speeds of 71.3–77.1 FPS. In contrast, appearance-based trackers introduced substantial computational overhead. BoT-SORT with appearance achieved the highest HOTA and IDF1 values; however, its processing speed decreased to approximately 30 FPS, while StrongSORT exhibited the highest computational burden, with a throughput of approximately 12 FPS.

The effect of appearance information was further examined by directly comparing BoT-SORT with its appearance module enabled and disabled. Under YOLOv8n, enabling appearance increased HOTA slightly from 72.3 to 72.5 and IDF1 from 79.5 to 80.1, while reducing IDSW from 24 to 19. However, processing speed decreased from 71.3 to 30 FPS. Under YOLOv11n, HOTA and IDF1 also improved only marginally with appearance enabled, while IDSW increased from 16 to 19 and processing speed decreased from 70.0 FPS to 30.6 FPS. These results indicate that appearance information provided limited accuracy improvements relative to its computational cost, particularly for long-duration lightweight tracking. Based on these findings, YOLOv8n combined with appearance-free BoT-SORT was selected as the final tracking configuration. Although BoT-SORT includes camera-motion compensation, its contribution is expected to be limited under the fixed-camera setting used in this study. Therefore, its improved performance is likely attributable primarily to enhanced motion modeling and data-association mechanisms.

### 3.3. Short Ground-Truth Segments: Frame Sampling, Interpolation and MTW-Repair

Using the selected tracking configuration (YOLOv8n + BoT-SORT without appearance) and ByteTrack as a fragmentation-prone reference tracker, stride values of 1, 2, 3, and 5 were compared under three processing conditions—raw tracker output, interpolation only (no MTW-Repair), and interpolation combined with MTW-Repair—using window parameters scaled proportionally to the stride (Section 2.4). This experiment aimed to determine the extent to which detection frequency could be reduced while maintaining frame-level tracking performance and to isolate the individual contributions of interpolation and MTW-Repair.

As shown in Table 4, raw HOTA decreased with frame sampling (e.g., ByteTrack: 71.9 at stride-1, 37.5 at stride-2, and 25.2 at stride-3). The raw outputs contain no predictions for skipped frames, resulting in missed detections when evaluated against full-frame ground truth. Frame sampling also reduces the temporal observations available for association. Applying interpolation alone recovered HOTA to within approximately 1–2 points of the stride-1 value at every stride. Interpolation estimates bounding boxes at skipped frames from the available observations; it does not recover the actual observations or guarantee accurate motion and identity estimates during those intervals. Adding MTW-Repair on top of interpolation produced a further, smaller improvement in association-related performance, particularly in AssA and IDSW, consistent with MTW-Repair acting on trajectory association without altering the underlying detections. For ByteTrack, IDSW decreased from 27 under the stride-1 raw condition to 14 after MTW-Repair. At stride-3, IDSW decreased from 6 after interpolation alone to 0 after interpolation plus MTW-Repair, while AssA increased from 85.2 to 87.1. At stride-1, MTW-Repair reduced IDSW by 13 for both trackers, from 27 to 14 for ByteTrack and from 24 to 11 for BoT-SORT. At strides 2, 3, and 5, the reductions relative to interpolation alone were smaller for BoT-SORT than for ByteTrack (Table 4). After interpolation and MTW-Repair, stride-3 retained HOTA and AssA values close to those at stride-1, whereas stride-5 yielded lower values for both trackers (Table 4). Stride-3 was used for subsequent long-duration experiments.

For relatively slow-moving animals such as cattle, sparse detection combined with interpolation can therefore substantially reduce computational requirements while maintaining comparable tracking performance. Effective throughput (detection FPS multiplied by stride) reached approximately 216–228 FPS at stride-3 for both trackers, roughly three times the stride-1 throughput, since only one-third of the frames required detection. To illustrate representative MTW-Repair merge events, Figure 5 shows four cases from the short ground-truth segments, each comprising a tracklet ending (red box), the fragmented successor tracklet before merging (green box), and the same detection re-labeled with the final merged identity (blue box) after MTW-Repair. Figure 5A,C shows stride-1 examples for ByteTrack and BoT-SORT, respectively, while Figure 5B,D shows stride-3 examples (with interpolation applied) for the same two trackers, drawn from the same source recording to allow direct visual comparison across strides.

### 3.4. Long-Duration Tracking: Fragmentation and Merge Distribution

ByteTrack and BoT-SORT were evaluated on the approximately 215 min long-duration recording (Table 5). Because frame-level ground truth was unavailable for this dataset, the total number of generated identities before and after MTW-Repair was used as the primary surrogate measure of long-duration tracking stability. Across all conditions, BoT-SORT generated substantially fewer total identities than ByteTrack, indicating reduced trajectory fragmentation, consistent with the short-GT results in Section 3.3. MTW-Repair reduced the total identity count from 3707 to 2129 for ByteTrack at stride-1 and from 1197 to 534 at stride-3, and from 1612 to 1126 for BoT-SORT at stride-1 and from 771 to 387 at stride-3. To characterize which temporal-gap range contributed most to these reductions, all accepted merges were classified according to the temporal window (short, medium, or long) in which they occurred (Table 6).

The distribution across windows differed markedly between trackers. For BoT-SORT at stride-1, long-window merges accounted for 67% of all merges (327/486), the highest proportion observed among all conditions, indicating that when BoT-SORT lost an identity, the resulting gap tended to be long rather than brief. This is consistent with its already stable short-term association (Section 3.2), such that most remaining fragmentation arose from extended occlusion events rather than momentary detection failures. For ByteTrack, merges were more evenly distributed between the medium and long windows at both strides, reflecting its higher baseline fragmentation rate across a wider range of gap durations. Under stride-3, medium- and long-window merges accounted for most accepted associations. Because the temporal-window boundaries differed between stride settings, these proportions were interpreted within each stride condition and were not treated as direct cross-stride comparisons of identical time ranges.

In addition to total identity counts, mean and median tracklet duration were computed directly from tracklet lifespans, without interpolation, providing an identity-continuity measure that is not affected by the gap-filling distortion described for the active-identity measure in the following paragraph. MTW-Repair consistently increased both the mean and median tracklet duration across all strides and both trackers (Figure 6), with the largest relative gain observed for ByteTrack at stride-3 (median duration increasing from 12.0 s to 49.0 s), indicating substantially reduced fragmentation under the adopted configuration. An active-identity count was examined during preliminary analysis but was excluded from the final evaluation because interpolation generated overlapping synthetic detections and inflated the estimated number of simultaneously active identities. Consequently, the long-duration evaluation focused on total identity count, predicted trajectory duration, and manual merge precision.

### 3.5. Merge Precision Verification

The overall merge precision was 84.0% (42/50; 95% confidence interval: 71.5–91.7%) (Table 7). By window size, precision was highest for the short window at 90.0% (9/10; 95% CI: 59.6–98.2%), followed by the medium window at 86.7% (13/15; 95% CI: 62.1–96.3%) and the long window at 80.0% (20/25; 95% CI: 60.9–91.1%). Although the confidence intervals for each category are relatively wide due to the division of the 50 samples into three window categories, the trend of monotonically decreasing precision from the short to the long window aligns with the loosening of spatial and temporal constraints as the gap widens, indicating that results from long-window merges require more cautious interpretation. Errors primarily manifested in two ways. First, merges involving large spatial distances were prone to incorrect associations, a problem that could be mitigated by applying stricter distance thresholds. Second, incorrect merges occurred even with small spatial distances when one animal vacated a location and another subsequently occupied it. This represents an inherent limitation that is difficult to resolve using distance information alone in dense environments and highlights the constraints of appearance-free tracking over long temporal gaps.

## 4. Discussion

Long-duration livestock tracking presents challenges that are not fully captured by short benchmark sequences [26]. In dense barn environments, frequent occlusion and repeated interactions among animals can cause progressive trajectory fragmentation and identity degradation, even when short-term tracking metrics remain high [27]. Practical monitoring systems therefore require not only accurate object association but also computational efficiency and stable identity continuity over extended periods. The present results demonstrate that these requirements can be partially achieved without relying on computationally intensive appearance information. In short ground-truth segments, MTW-Repair reduced identity switches while maintaining HOTA at every sampled stride, indicating that the recovered associations were largely reliable. Although direct IDSW evaluation was not possible for the ground-truth-free long-duration recording, the total-identity and tracklet-duration measures provided indirect, ground-truth-independent evidence of reduced trajectory fragmentation.

These findings also highlight the limitations of appearance-based tracking in dense Hanwoo cattle environments. A previous study integrated appearance modeling, a non-linear Kalman filter [12], and trajectory recovery to achieve strong association performance; however, the appearance model introduced substantial computational overhead. In the present study, enabling appearance information improved HOTA by 0.2 points and IDF1 by 0.6 points with YOLOv8n, while reducing processing speed from 71.3 to 30 FPS. This limited improvement indicates that the evaluated off-the-shelf person-pretrained OSNet configuration provided insufficient additional discrimination under the present Hanwoo cattle barn conditions [20]. Therefore, under the evaluated conditions, appearance-free tracking provided a more efficient balance between accuracy and computational cost.

The comparison between ByteTrack and BoT-SORT further indicates that long-duration tracking performance depends strongly on trajectory fragmentation control rather than short-sequence benchmark metrics alone. Although the two trackers showed similar performance on short annotated segments, BoT-SORT generated substantially fewer identities over the 215 min recording and exhibited longer tracklet durations. These results suggest that conventional MOT metrics, including HOTA, MOTA, and IDF1, while valuable for short annotated sequences, may not fully reflect fragmentation accumulation over extended monitoring periods. The stride-dependent behavior of MTW-Repair observed for BoT-SORT, including 486 accepted merges at stride-1 and 384 at stride-3 in the long-duration recording, further demonstrates that its effect depends jointly on the fragmentation characteristics of the underlying tracker and the frame-sampling rate. MTW-Repair provided larger corrections under conditions with greater baseline fragmentation, particularly for ByteTrack, whereas its additional effect was smaller when the underlying tracklets were already relatively stable. Thus, MTW-Repair should be considered a tracker-independent post-processing strategy whose effectiveness scales with the degree of trajectory fragmentation present, rather than a tracker-specific enhancement.

The frame-sampling experiments demonstrate the feasibility of a lightweight temporal strategy for long-duration livestock monitoring. Frame sampling reduces both the detection workload and the temporal observations available to the tracker. Interpolation improved full-frame evaluation scores by estimating bounding boxes at skipped frames, but these estimates should not be interpreted as equivalent to processing every frame. MTW-Repair subsequently addressed fragmented identity assignments in the resulting trajectories. Stride-3 reduced the detection workload to one-third while maintaining comparable HOTA and MOTA values. Since object detection is typically the dominant computational cost in tracking-by-detection video monitoring pipelines [13,14], combining sparse detection with lightweight trajectory refinement provides a practical approach for improving scalability in continuous livestock observation.

### Limitations of the Study

Several limitations should be acknowledged. First, the temporal windows, filtering thresholds, and merge-score weights were selected empirically. These choices may be overly adapted to the evaluated recordings, and their generalizability has not been established through independent validation across farms and camera configurations. The current spatial and temporal criteria also depend on image resolution and frame rate. Camera angle and perspective affect apparent displacement and bounding-box size, so transferring the method to another installation may require parameter recalibration. Second, evaluation was limited to a single fixed camera at one farm, with seven animals, four short annotated clips, and one long-duration recording. This design does not establish performance in larger herds, different housing layouts, or other viewing conditions. Frame sampling also reduces the observations available for tracking. Faster movement or abrupt changes in direction may increase association errors and reduce the accuracy of linear interpolation, particularly during occlusion. Third, false merges can connect trajectories belonging to different animals, reducing the total identity count while introducing incorrect identity associations. Such errors may distort subsequent estimates of individual movement and behavior. Manual verification yielded a merge precision of 84.0% in 50 sampled ByteTrack merge events, but this estimate does not establish merge accuracy across the entire recording or for BoT-SORT. Merge precision also does not measure the proportion of true identity continuations successfully recovered. Finally, complete ground truth was unavailable for the long-duration recording, preventing measurement of long-duration identity recall and comprehensive verification of individual identity continuity. Total identity counts and tracklet durations describe the predicted trajectories but cannot establish that identities remain correct throughout the recording. The active-identity measure was excluded because interpolation distorted the estimated number of simultaneously active identities. Extended identity gaps remain unresolved, and further evaluation requires longer identity-annotated recordings, larger herds, independent camera settings, and explicit assessment of parameter sensitivity and false-merge effects.

## 5. Conclusions

This study evaluated a lightweight tracking framework combining YOLOv8n, appearance-free BoT-SORT, frame sampling, interpolation, and MTW-Repair using a single fixed RGB camera at one commercial farm housing seven Hanwoo cattle under intensive housing conditions. Stride-3 sampling reduced the number of frames requiring detection by approximately two-thirds. In the short annotated clips, interpolation improved full-frame tracking scores, and MTW-Repair reduced identity switches. In the approximately 215 min recording, MTW-Repair reduced predicted identity counts and increased tracklet duration for both ByteTrack and BoT-SORT. Manual verification of 50 randomly sampled ByteTrack merges from the stride-3 output yielded a precision of 84.0%. These results support computationally efficient tracklet consolidation under the evaluated conditions. However, the long-duration surrogate measures do not establish persistent individual identity accuracy, and performance across farms, larger herds, and different camera configurations requires further validation.

## Figures and Tables

**Figure 1:**
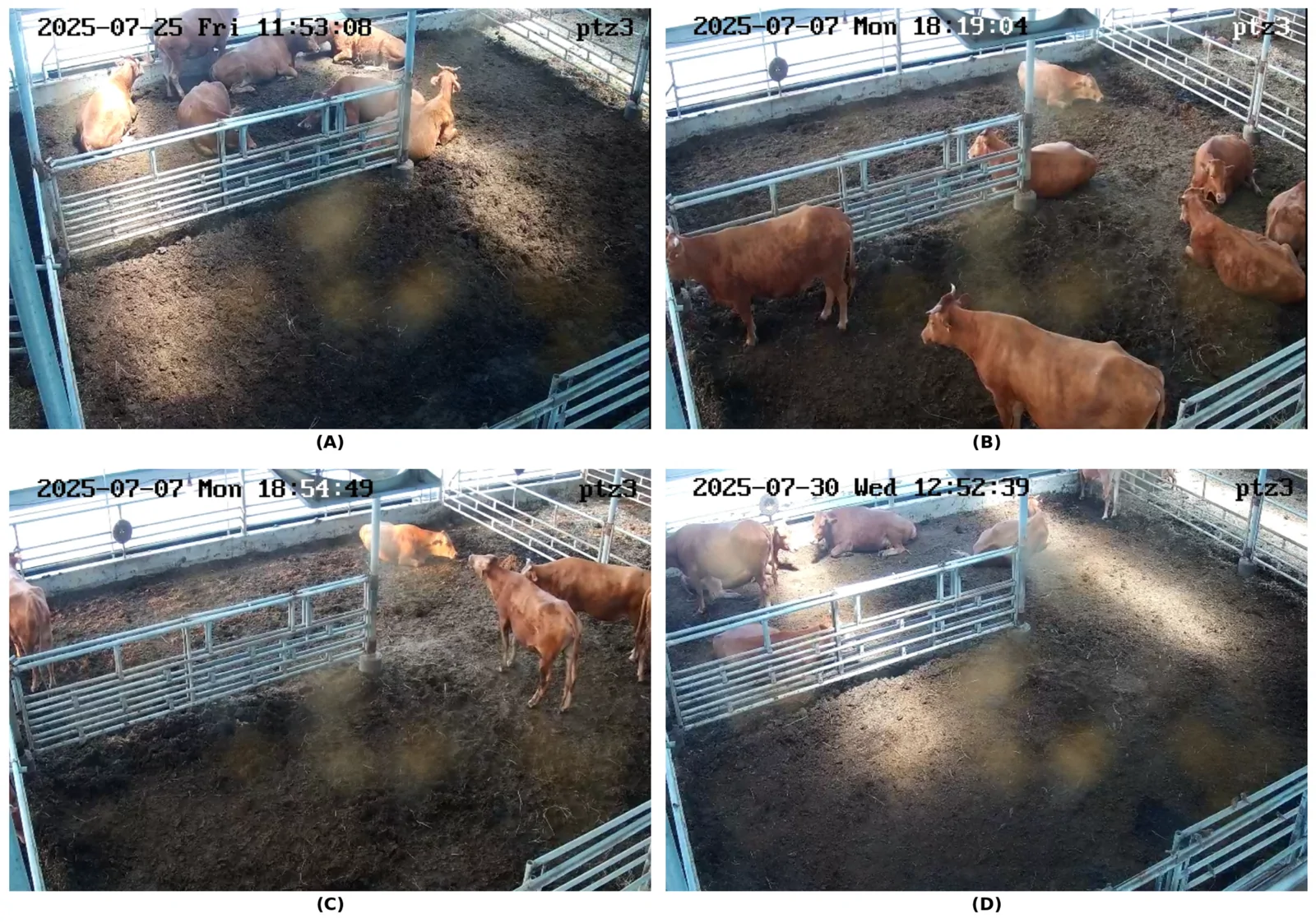
Representative frames from the four source recordings (**A**–**D**) used in this study. All videos were collected at a commercial Hanwoo cattle farm in Anseong, Republic of Korea, using a fixed ceiling-mounted CCTV camera.

**Figure 2:**
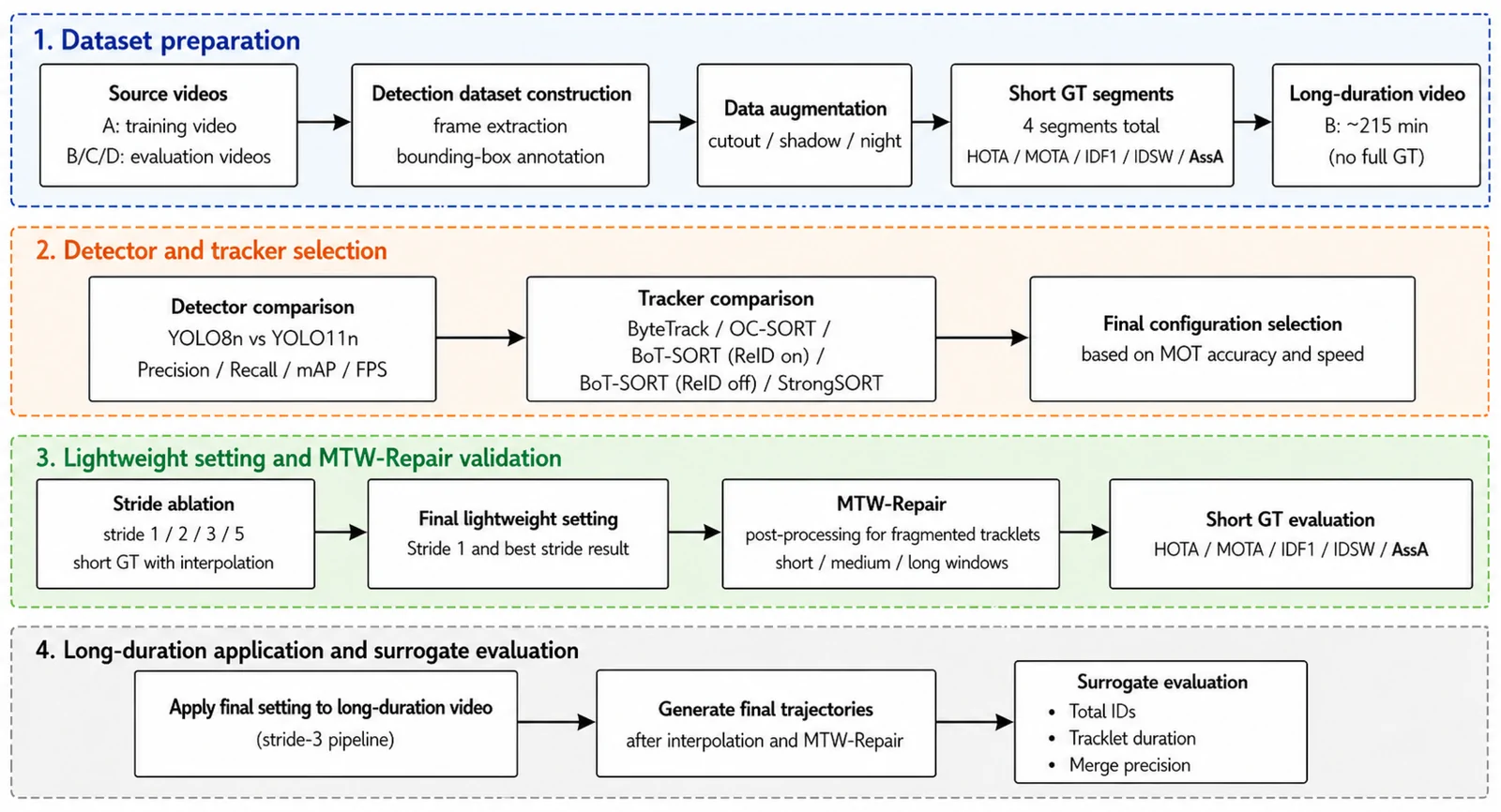
Overview of the proposed lightweight long-duration tracking framework: (1) dataset preparation; (2) detector and tracker selection; (3) frame-sampling and MTW-Repair validation; and (4) long-duration application and surrogate evaluation.

**Figure 3:**
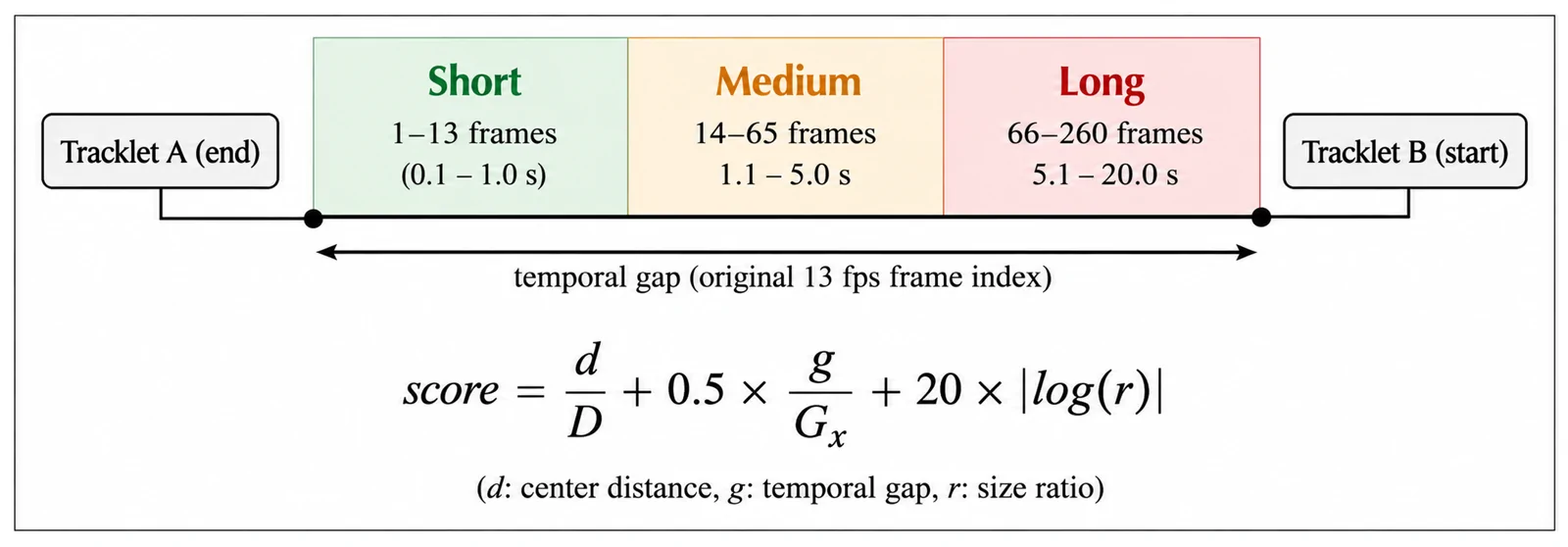
Base gap-dependent temporal windows used in MTW-Repair at stride-1. The temporal gap between the end of Tracklet A and the start of Tracklet B determines the applicable short, medium, or long window. Candidate pairs satisfying the corresponding filtering conditions are prioritized using the merge score, with lower-scoring pairs merged first through greedy one-to-one matching. Exact distance, implied-speed, and bounding-box size-ratio thresholds are provided in Table 1.

**Figure 4:**
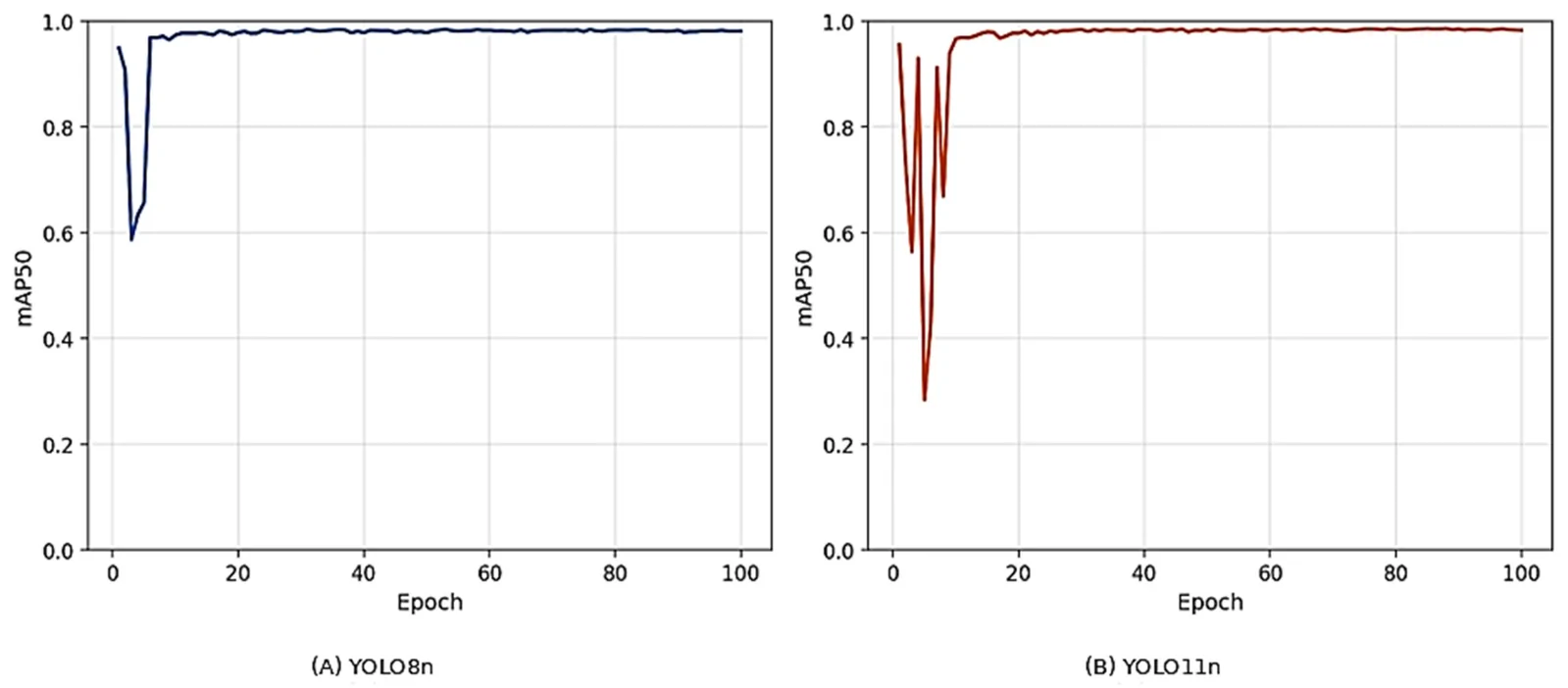
Training mAP50 curves over 100 epochs for (**A**) YOLOv8n and (**B**) YOLOv11n. Both models showed rapid improvement during the early epochs and stable convergence in the later epochs.

**Figure 5:**
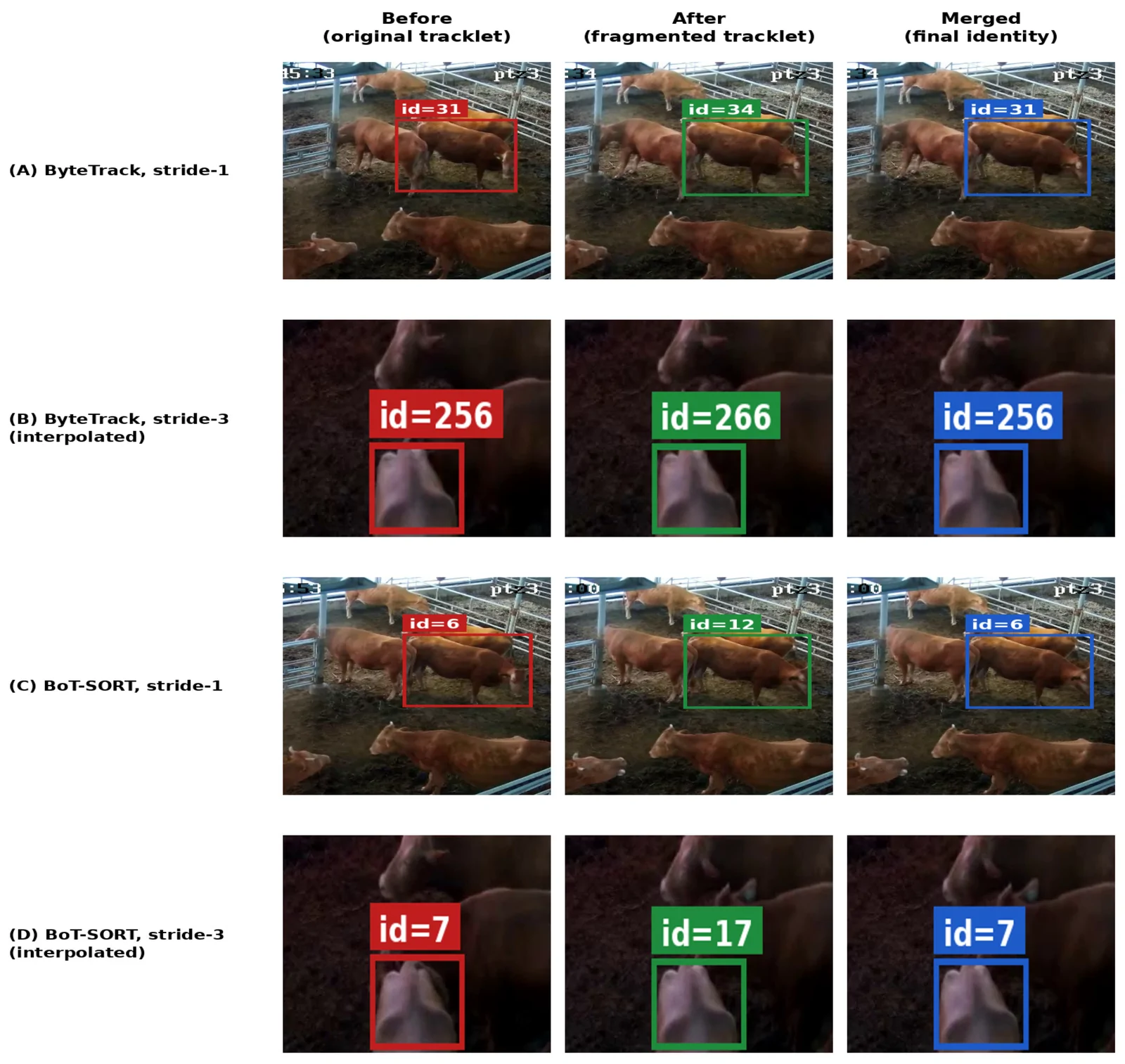
Representative MTW-Repair merge events on the short ground-truth segments. (**A**) ByteTrack, stride-1; (**B**) ByteTrack, stride-3 (interpolated); (**C**) BoT-SORT, stride-1; (**D**) BoT-SORT, stride-3 (interpolated). In each panel, the tracklet ending (red box, original identity) and the fragmented successor tracklet (green box, newly assigned identity) are shown before merging, followed by the same detection re-labeled with the final merged identity (blue box) after MTW-Repair, connected by arrows to indicate the merge direction.

**Figure 6:**
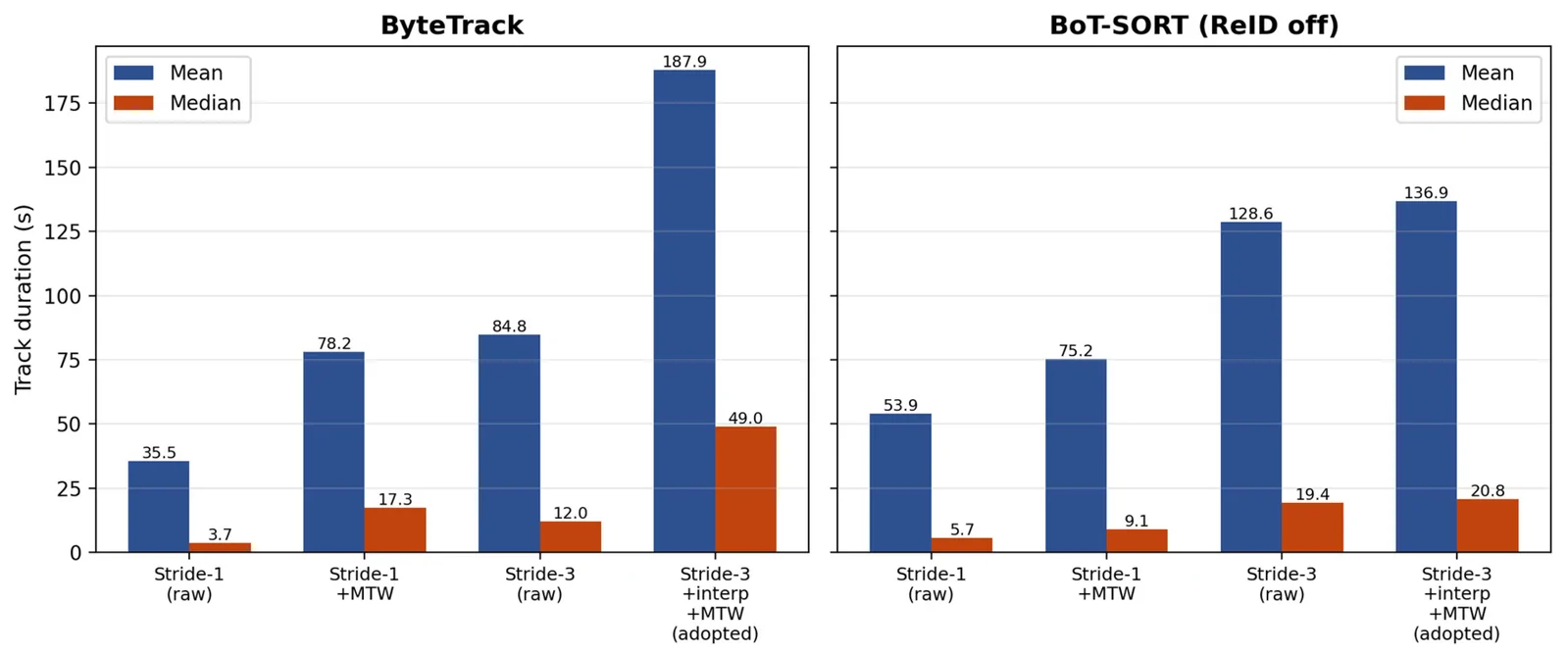
Mean and median tracklet duration on the 215 min long-duration recording for ByteTrack and BoT-SORT (ReID off), computed directly from tracklet lifespans without interpolation, across raw, stride-1 + MTW-Repair, stride-3 (raw), and adopted stride-3 + interpolation + MTW-Repair configurations.

**Table 1:** Base temporal-window and candidate-filtering parameters of MTW-Repair defined at stride-1 using the original 13 fps frame index. Stride-specific implementation boundaries were obtained by multiplying the base frame ranges by the stride value. The parameters were selected empirically, and a sample-based manual verification of accepted ByteTrack merges under stride-3 is reported in Section 3.5.

Window	Temporal Gap (Frames, 13 fps)	Temporal Gap (s)	Distance Threshold (px)	Speed Threshold (px/Frame)	Size-Ratio Range
Short	1–13	0.08–1.00	60	10	0.55–1.75
Medium	14–65	1.08–5.00	110	7	0.60–1.65
Long	66–260	5.08–20.00	170	4	0.65–1.55

**Table 2:** Detection performance of YOLOv8n and YOLOv11n on the Hanwoo cattle validation set after 100 training epochs at an input resolution of 640 × 640 pixels. mAP50 and mAP50–95 denote mean average precision at an intersection-over-union threshold of 0.50 and across thresholds of 0.50–0.95, respectively. FPS denotes detector inference speed in frames per second.

Detector	Precision	Recall	mAP50	mAP50-95	FPS
YOLOv8n	0.971	0.961	0.981	0.823	91.70
YOLOv11n	0.977	0.965	0.986	0.824	133.50

**Table 3:** Tracking performance of five tracker configurations combined with YOLOv8n and YOLOv11n detectors.

Detector	Tracker	HOTA	AssA	MOTA	IDF1	IDSW	FPS
YOLOv8n	ByteTrack	71.9	83.1	69.1	78.7	27	77.1
YOLOv8n	OC-SORT	71.0	81.7	67.6	79.4	39	74
YOLOv8n	BoT-SORT (ReID off)	72.3	82.6	70.9	79.5	24	71.3
YOLOv8n	BoT-SORT (ReID on)	72.5	82.6	71.1	80.1	19	30
YOLOv8n	StrongSORT	71.3	81.8	67.1	79.4	38	12
YOLOv11n	ByteTrack	70.6	84.3	63.2	75.7	25	74.7
YOLOv11n	OC-SORT	70.2	80.0	64.8	77.6	28	69.7
YOLOv11n	BoT-SORT (ReID off)	69.5	81.5	64.8	75.3	16	70.0
YOLOv11n	BoT-SORT (ReID on)	70.4	83.7	64.9	76.5	19	30.6
YOLOv11n	StrongSORT	68.5	73.4	64.1	74.4	52	11.9

**Table 4:** Effect of stride-based frame sampling, interpolation, and MTW-Repair on tracking performance (YOLOv8n + ByteTrack and BoT-SORT (ReID off)). Stride-specific MTW-Repair window boundaries were obtained by multiplying the base stride-1 frame ranges reported in Table 1 by the corresponding stride value. The final configuration (interpolation + MTW-Repair, shaded) is used for all subsequent long-duration experiments at stride-3.

Tracker	Stride	Condition	HOTA	AssA	MOTA	IDF1	IDSW	Tracking FPS	Effective FPS
ByteTrack	1	Raw	71.9	83.1	69.1	78.7	27	77.1	77.1
		MTW-Repair	73.7	87.3	69.2	82.5	14	77.1	77.1
	2	Raw	37.5	43.1	34.7	52.8	14	78.7	157.3
		Interpolation only	72.8	85.3	69.5	81.2	12	78.7	157.3
		Interpolation + MTW-Repair	73.7	87.3	69.5	83.4	4	78.7	157.3
	3	Raw	25.2	28.8	23.1	39.2	6	75.9	227.8
		Interpolation only	72.7	85.2	69.5	81.6	6	75.9	227.8
		Interpolation + MTW-Repair	73.5	87.1	69.5	83.4	0	75.9	227.8
	5	Raw	14.9	16.7	13.8	25.1	8	71.3	355.2
		Interpolation only	70.3	80.9	69.0	79.5	8	71.3	355.2
		Interpolation + MTW-Repair	70.7	82.0	69.0	80.5	5	71.3	355.2
BoT-SORT (ReID off)	1	Raw	72.3	82.6	70.9	79.5	24	71.3	71.3
		MTW-Repair	74.0	86.8	71.1	83.6	11	71.3	71.3
	2	Raw	37.9	43.6	35.5	53.5	12	73.6	147.2
		Interpolation only	73.7	86.1	70.9	82.5	14	73.6	147.2
		Interpolation + MTW-Repair	73.8	86.4	70.9	82.8	10	73.6	147.2
	3	Raw	25.6	29.4	23.7	39.8	6	72.2	216.7
		Interpolation only	74.0	86.9	71.0	83.0	7	72.2	216.7
		Interpolation + MTW-Repair	74.0	86.9	71.0	83.1	5	72.2	216.7
	5	Raw	15.3	17.1	14.3	25.8	6	69.8	348.8
		Interpolation only	72.5	83.4	71.2	81.7	6	69.8	348.8
		Interpolation + MTW-Repair	72.5	83.4	71.2	81.7	5	69.8	348.8

**Table 5:** Total identity counts before and after MTW-Repair on the 215 min long-duration recording. The adopted configuration (stride-3 + interpolation + MTW-Repair, shaded) applies interpolation prior to MTW-Repair, consistent with the short-GT pipeline (Section 3.3), and uses window parameters scaled proportionally to the stride (Table 1).

Tracker	Configuration	Total IDs (Raw)	Total IDs (Final)
ByteTrack	Stride-1 + MTW-Repair	3707	2129
ByteTrack	Stride-3 + interpolation + MTW-Repair (adopted)	1197	534
BoT-SORT (ReID off)	Stride-1 + MTW-Repair	1612	1126
BoT-SORT (ReID off)	Stride-3 + interpolation + MTW-Repair (adopted)	771	387

**Table 6:** Breakdown of MTW-Repair merges by temporal window on the 215 min long-duration recording. Long-window merges account for the largest share at stride-1 for both trackers (52% and 67%, respectively), while medium-window merges become more prominent at stride-3 following interpolation.

Tracker	Configuration	Short	Medium	Long	Total Merges
ByteTrack	Stride-1 + MTW-Repair	46	715	817	1578
ByteTrack	Stride-3 + interpolation + MTW-Repair (adopted)	40	309	314	663
BoT-SORT (ReID off)	Stride-1 + MTW-Repair	28	131	327	486
BoT-SORT (ReID off)	Stride-3 + interpolation + MTW-Repair (adopted)	13	159	212	384

**Table 7:** Manual verification of MTW-Repair merge precision for 50 randomly sampled ByteTrack merge events at stride 3. Precision is reported with Wilson 95% confidence intervals.

Category	Precision	Correct/Total	95% CI
Overall	84.0%	42/50	71.5–91.7%
Short window	90.0%	9/10	59.6–98.2%
Medium window	86.7%	13/15	62.1–96.3%
Long window	80.0%	20/25	60.9–91.1%

## Data Availability

Upon reasonable request, the datasets of this study can be made available from the corresponding author.

## Abbreviations

The following abbreviations are used in this manuscript:

AssA	Association Accuracy
FPS	Frames Per Second
GPS	Global Positioning System
GT	Ground Truth
HOTA	Higher Order Tracking Accuracy
HSV	Hue, Saturation, Value
IDF1	Identification F1 Score
IDFN	Identity False Negative
IDFP	Identity False Positive
IDSW	Identity Switch
IDTP	Identity True Positive
MOT	Multi-Object Tracking
MOTA	Multi-Object Tracking Accuracy
MTW-Repair	Multi-Temporal Window Repair
OC-SORT	Observation-Centric SORT
OSNet	Omni-Scale Network
ReID	Re-identification
SGD	Stochastic Gradient Descent
YOLOv11n	YOLOv11 Nano
YOLOv8n	YOLOv8 Nano
